# Comparative Chloroplast Genomics of *Gossypium* Species: Insights Into Repeat Sequence Variations and Phylogeny

**DOI:** 10.3389/fpls.2018.00376

**Published:** 2018-03-21

**Authors:** Ying Wu, Fang Liu, Dai-Gang Yang, Wei Li, Xiao-Jian Zhou, Xiao-Yu Pei, Yan-Gai Liu, Kun-Lun He, Wen-Sheng Zhang, Zhong-Ying Ren, Ke-Hai Zhou, Xiong-Feng Ma, Zhong-Hu Li

**Affiliations:** ^1^State Key Laboratory of Cotton Biology, Institute of Cotton Research, Chinese Academy of Agricultural Sciences, Anyang, China; ^2^Key Laboratory of Resource Biology and Biotechnology in Western China, Ministry of Education, College of Life Sciences, Northwest University, Xi’an, China

**Keywords:** chloroplast genome, divergent hotspot, *Gossypium*, phylogeny, repeat sequence

## Abstract

Cotton is one of the most economically important fiber crop plants worldwide. The genus *Gossypium* contains a single allotetraploid group (AD) and eight diploid genome groups (A–G and K). However, the evolution of repeat sequences in the chloroplast genomes and the phylogenetic relationships of *Gossypium* species are unclear. Thus, we determined the variations in the repeat sequences and the evolutionary relationships of 40 cotton chloroplast genomes, which represented the most diverse in the genus, including five newly sequenced diploid species, i.e., *G. nandewarense* (C_1-n_), *G. armourianum* (D_2-1_), *G. lobatum* (D_7_), *G. trilobum* (D_8_), and *G. schwendimanii* (D_11_), and an important semi-wild race of upland cotton, *G. hirsutum* race *latifolium* (AD_1_). The genome structure, gene order, and GC content of cotton species were similar to those of other higher plant plastid genomes. In total, 2860 long sequence repeats (>10 bp in length) were identified, where the F-genome species had the largest number of repeats (*G. longicalyx* F_1_: 108) and E-genome species had the lowest (*G. stocksii* E_1_: 53). Large-scale repeat sequences possibly enrich the genetic information and maintain genome stability in cotton species. We also identified 10 divergence hotspot regions, i.e., *rpl33-rps18, psbZ-trnG* (*GCC*), *rps4-trnT* (*UGU*), *trnL* (*UAG*)*-rpl32, trnE* (*UUC*)*-trnT* (*GGU*), *atpE, ndhI, rps2, ycf1*, and *ndhF*, which could be useful molecular genetic markers for future population genetics and phylogenetic studies. Site-specific selection analysis showed that some of the coding sites of 10 chloroplast genes (*atpB, atpE, rps2, rps3, petB, petD, ccsA, cemA, ycf1*, and *rbcL*) were under protein sequence evolution. Phylogenetic analysis based on the whole plastomes suggested that the *Gossypium* species grouped into six previously identified genetic clades. Interestingly, all 13 D-genome species clustered into a strong monophyletic clade. Unexpectedly, the cotton species with C, G, and K-genomes were admixed and nested in a large clade, which could have been due to their recent radiation, incomplete lineage sorting, and introgression hybridization among different cotton lineages. In conclusion, the results of this study provide new insights into the evolution of repeat sequences in chloroplast genomes and interspecific relationships in the genus *Gossypium*.

## Introduction

Cotton is one of the most economically important fiber crop plants throughout the world (Wendel, 1989; Ruan et al., 2003). The genus *Gossypium* L. comprises about 53 species, four of which have cultivated forms with two diploids and two allotetraploids (Fryxell, 1969, 1978; Wendel and Cronn, 2003; Grover et al., 2007; Wendel et al., 2009; Wendel and Grover, 2015). Recently, some new species have been discovered and characterized (Stewart et al., 2015; Gallagher et al., 2017). Divergence analysis based on DNA molecular markers suggests that the major diploid branches of the cotton genus diverged about 7–11 million years ago (Senchina et al., 2003; Wendel et al., 2009; Wendel and Grover, 2015; Chen et al., 2016, 2017a). Subsequently, the ancestor of cotton diversified into ∼46 diploid species (divided into eight genome groups designated as A–G and K) and 7 allotetraploid species designated as the AD genome (Senchina et al., 2003; Wendel et al., 2010; Grover et al., 2015; Wendel and Grover, 2015; Chen et al., 2017a,b). In general, it is considered that the polyploid clade originated circa 1–2 million years ago, possibly due to transoceanic dispersal events involving an African-Asian A-genome species that subsequently hybridized with a New World D-genome species (Wendel, 1989; Adams and Wendel, 2004; Wendel and Grover, 2015; Chen et al., 2016, 2017a,b).

Therefore, cotton species provide an excellent and fascinating model system for studying polyploidization, migration, and biogeographic dispersal among different continents (Fryxell, 1969, 1978; Wendel, 1989; Wendel and Grover, 2015; Chen et al., 2016, 2017a,b). Recently, the whole nuclear genomes have been reported for the model diploid D-genome (Paterson et al., 2012; Wang et al., 2012; Liu et al., 2013), A-genome (Li et al., 2014), and allopolyploid AD genome species *G. barbadense* (Liu et al., 2015; Yuan et al., 2015) and *G. hirsutum* (Li et al., 2015; Zhang et al., 2015). These newly released genomes provide useful molecular genetic resources for studying the origin and evolution of cotton species. Studies have identified the dual domestication and origin of cultivated cotton species (*G. hirsutum* and *G. barbadense*) based on large-scale genome variations (Fang et al., 2017). In addition, the major phylogenetic framework of the cotton genus was established based on available morphological evidence and molecular biology data sets (Fryxell, 1969, 1978; Wendel, 1989; Wendel and Albert, 1992; Khan et al., 2000; Cronn et al., 2002; Wendel and Cronn, 2003; Grover et al., 2008; Wendel and Grover, 2015; Chen et al., 2016, 2017a,b).

In recent years, due to the rapid development of next generation sequencing, the maternally inherited chloroplast genomes have largely been assembled and used to study the phylogeny and evolutionary relationships in the cotton genus (Ibrahim et al., 2006; Lee et al., 2006; Xu et al., 2012; Chen et al., 2016, 2017a). In general, plant chloroplast genomes are circular DNA molecule structures ranging from 115 to 165 kb in size (Wolfe et al., 1987; Jansen et al., 2005), with a highly conserved quadripartite structure comprising two inverted repeats (IRa/b) separated by a large single copy (LSC) region and a small single copy (SSC) (Raubeson and Jansen, 2005; Wicke et al., 2011). The highly conserved characteristics and relatively independent evolutionary properties of plastomes make them useful for the rapid analysis of species evolution and phylogenetic relationships (Jansen et al., 2007; Parks et al., 2009; Wang et al., 2013; Chen et al., 2014). For example, studies based on whole plastome sequence variations identified the six major *Gossypium* genetic clades comprising A+AD, F, E, D, B, and C+G+K genome groups. Evidence also suggests that the divergence of cotton species occurred rapidly in the recent past (Cronn et al., 2002; Chen et al., 2017a). In addition, it has been shown that nucleotide substitution mutations have occurred more frequently than variations due to insertions and/or deletions in the *Gossypium* chloroplast genomes (Chen et al., 2016). The differences in the size of cotton plastomes are largely due to variations in their LSC regions (Chen et al., 2017a). However, previous studies only sampled a small number of cotton species and the evolutionary relationships among many of the *Gossypium* lineage branches are still unknown. In addition, variations in the repeat sequences in the cotton chloroplast genomes remain unexplored.

In the current study, we collected 40 cotton chloroplast genomes representing the highest diversity known at present in the genus *Gossypium*, including five newly sequenced diploid species comprising *G. nandewarense* (C_1-n_), *G. armourianum* (D_2-1_), *G. lobatum* (D_7_), *G. trilobum* (D_8_), and *G. schwendimanii* (D_11_), and a semi-wild race of upland cotton, *G. hirsutum* race *latifolium* (AD_1_). The aims of this study were as follows: (1) to examine the variations in the repeat sequences in chloroplast genomes; (2); to detect divergence hotspots in plastid genomes; (3) to analyze protein sequence evolution in the coding regions; and (4) to reconstruct the phylogenetic relationships of the major lineages in the genus *Gossypium*.

## Materials and Methods

### Plant Sampling and DNA Extraction

Fresh leaves of five diploid species comprising *G. nandewarense* (C_1-n_), *G. armourianum* (D_2-1_), *G. lobatum* (D_7_), *G. trilobum* (D_8_), and *G. schwendimanii* (D_11_), and a semi-wild race of upland cotton, *G. hirsutum* race *latifolium* (AD_1_), were collected from the National Wild Cotton Nursery in Sanya, China, and the leaves were dried with silica gel. High-quality genomic DNA was isolated using a modified CTAB method (Li et al., 2013). The quality of the DNA was examined using agarose gel electrophoresis. DNA with a final concentration >30 ng μL^-1^ was selected for next generation high throughput sequencing.

### DNA Library Construction, Sequencing, Chloroplast Genome Assembly, and Annotation

Using the isolated high quality DNA, we first constructed a paired end library with an insert size of 350 bp using TruSeq DNA sample preparation kits. Subsequently, we sequenced at least 4 GB of clean data for each cotton species with an average read length of 125 bp (**Supplementary Table S1**). All of the sequencing reactions were conducted on the Illumina Hiseq 2500 platform at Biomarker Technologies Co., Ltd. (Beijing, China). The raw reads obtained were quality trimmed using the program NGSQCtoolkit v2.3.3 (Patel and Jain, 2012). We used the reference-guided assembly method to reconstruct the plastid genomes with MIRA v4.0.2 (Chevreux et al., 2004) and MITObim-master (Hahn et al., 2013). In this analysis, we used the chloroplast genome of the closely related species G. *hirsutum* (AD_1_) (NC_007944) as the reference sequence. In addition, four small gaps and ambiguous sequences were verified by first generation Sanger sequencing, three of which comprising *trnE* (*UUC*)-*trnT* (*GGU*), *ndhF-trnN* (*GUU*), and *ndhF-trnN* (*GUU*) were located in the intergenic regions of the single copy regions, whereas *rpl16 intron* was located in the intron region. The primer pairs were generated using Primer3 (**Supplementary Table S2**) (Untergrasser et al., 2012). Chloroplast DNA was annotated using DOGMA (Wyman et al., 2004) with manual adjustments. Finally, we identified tRNA genes using DOGMA and tRNAscan-SE search server (Lowe and Eddy, 1997). All of the newly generated sequences were submitted to GenBank (accession numbers MG891801–MG891803, MG800784, MG779276, and MG800783). The circular *Gossypium* chloroplast genome maps were drawn using Organellar Genome DRAW v1.1 (OGDRAW) (Lohse et al., 2013).

### Characterization of Repeat Sequences

We calculated the repeat types and their numbers in all the cotton plastid genomes. We detected tandem repeat sequences (>10 bp in length) using Tandem Repeats Finder (Benson, 1999). REPuter was used to visualize dispersed and palindromic repeats with a minimum repeat size of 30 bp, edit distances of less than 3 bp, and two repeat copies with at least 90% similarity (Kurtz et al., 2001). We also calculated the simple sequence repeats (SSRs) using the Perl script MISA (Thiel et al., 2003) with a motif size of 1–6 nucleotides and thresholds of ten, five, four, three, three, and three repeat units for mono-, di-, tri-, tetra-, penta-, and hexanucleotide SSRs, respectively.

### Sequence Divergence Analysis

In order to detect sequence divergence in the cotton chloroplast genomes, we randomly selected eight of the available diploid species (one representative for each of the eight genomes: A–G and K) and one allotetraploid AD species, as well as the newly sequenced six cotton species. Alignments of the 15 chloroplast genomes were visualized using mVISTA with *G. hirsutum* as a reference (Frazer et al., 2004). The percentages of nucleotide variation were calculated according to the method of Zhang et al. (2011). In addition, in order to identify microstructural mutations in all the *Gossypium* plastomes, we determined the nucleotide substitution sites using MEGA 5.0 (Tamura et al., 2011), and indels (insertion/deletion) were detected manually in the cotton chloroplast genomes.

### Protein Sequence Evolution Analysis

In order to detect the sites under selection in the protein-coding genes in cotton plastid genomes, the non-synonymous (dN) and synonymous (dS) nucleotide substitution rates and their ratio (ω = dN/dS) were calculated using the Codeml program in the PAML4.7 package (options were set to seqtype = 1, model = 0, NSsites = 0, 1, 2, 3, 7, 8 in the codeml.ctl file) (Yang and Nielsen, 2002; Yang et al., 2005). PAML analyses were conducted in the “user tree” mode. The maximum likelihood (ML) phylogenetic evolutionary tree was obtained based on the complete chloroplast genomes using RAxML (Stamatakis, 2006). We employed site-specific models to analyze the selection pressure on 78 common protein-coding genes shared by all of the cotton genomes. This model allowed the ω ratio to vary among sites with a fixed ω ratio in all the evolutionary branches. We compared three sets of assumptions: M1 (nearly neutral) vs. M2 (positive selection), M7 (beta) vs. M8 (beta and ω), and M0 (one-ratio) vs. M3 (discrete). We used the log-likelihood ratio test (LRT) (Yang and Nielsen, 2002) and Akaike’s information criterion (AIC) scores (Akaike, 1998; Aho et al., 2014) to estimate the quality of each model. The sequences of the genes under positive selection were translated into amino acid sequences and submitted to SWISS-MODEL^1^ to build three-dimensional structures. The locations of the amino acids in the RuBisCO molecule structure were examined using DeepView – The Swiss-PDBViewer v.3.7 (Guex and Peitsch, 1997).

### Phylogenetic Analysis

In order to determine the evolutionary relationships among cotton species, 40 available plastid genome sequences from *Gossypium* species were used to construct the molecular evolutionary tree, where *Hibiscus syriacus* and *Theobroma cacao* were used as outgroups (Chen et al., 2016, 2017a). The molecular phylogenetic analysis was conducted based on the following two data partitions: (1) the complete chloroplast genomes and (2) the protein-coding sequences. All of the chloroplast DNA sequences were first aligned using MAFFT (Katoh and Standley, 2013) and MEGA 5.0 (Tamura et al., 2011). We then used Modeltest v3.7 (Posada and Crandall, 1998) and the AIC values to detect the most appropriate molecular evolutionary model. Finally, ML analysis was conducted using RAxML v7.2.8 (Stamatakis, 2006) with the best model comprising GTR+G based on 1000 bootstrap replicate tests.

## Results

### Molecular Features of Plastomes

The newly sequenced plastid genomes from six *Gossypium* species ranged in size from 159,677 bp for *G. nandewarense* to 160,347 bp for *G. hirsutum* race *latifolium*. These genomes had a quadripartite molecule structure where the same regions had similar lengths. The gene order and composition were identical in the six species examined, and they were also similar to other previously published *Gossypium* chloroplast genomes. The coding sequence lengths of the six *Gossypium* chloroplast genomes ranged from 78,528 bp (*G. hirsutum* race *latifolium*) to 78,696 bp (*G. lobatum*). The length of LSC ranged from 88,284 bp (*G. nandewarense*) to 88,848 bp (*G. hirsutum* race *latifolium*). The SSC and IR sizes ranged from 20,233 bp (*G. trilobum*) to 20,318 bp (*G. schwendimanii*) and from 25,550 bp (*G. lobatum*) to 25,606 bp (*G. hirsutum* race *latifolium*), respectively (**Table 1**). All six chloroplast genomes contained 112 unique functional genes, with 78 protein-coding genes, 4 ribosomal RNA genes (*rrn23, rrn4.5, rrn5*, and *rrn16*), and 30 tRNA genes. Eighteen duplicated genes were located in IR regions, and thus each plastid genome harbored 130 genes in total. Eighteen genes contained intron sequences, where three genes comprising *clpP, rps12*, and *ycf3* had two introns, whereas the others each had only one intron (*arpF, ndhA, ndhB, petB, petD, rpoC1, rpl2, rpl16, rps16, trnA-UGC, trnG-UCC, trnI-GAU, trnK-UUU, trnL-UAA*, and *trnV-UAC)* (**Supplementary Tables S3, S4**). Furthermore, the *matK* gene was located within the *trnK-UUU* intron in the cotton chloroplast genomes. The GC content of each species was very similar in the same sequence region or complete plastid genome, but the GC content of the IR region was clearly higher than that of the other plastid DNA regions (**Table 1** and **Supplementary Figure S1**). The overall GC content of the cotton plastomes ranged from 37.1 to 37.4%, where it was highest in the E-genome group at 37.4% and lowest in the C-genome group (*G. sturtianum*) at 37.1%, while it was about 37.3% in the B and D genome groups, and approximately 37.2% in the other genome groups (**Supplementary Tables S3, S4**).

**Table 1:** Characteristics of chloroplast genomes in six *Gossypium* species.

Genome features	*G. armourianum*	*G. hirsutum* race *latifolium*	*G. nandewarense*	*G. trilobum*	*G. lobatum*	*G. schwendimanii*
Size (bp)	160080	160347	159677	160142	160205	160199
LSC length (bp)	88657	88848	88284	88735	88811	88779
SSC length (bp)	20241	20287	20241	20233	20294	20318
IR length (bp)	25591	25606	25576	25587	25550	25551
Coding (bp)	78612	78528	78531	78552	78696	78681
Non-coding (bp)	81468	81819	81146	81590	81509	81518
Number of genes	130	130	130	130	130	130
Protein-coding genes	85	85	85	85	85	85
tRNA genes	37	37	37	37	37	37
rRNA genes	8	8	8	8	8	8
Overall GC content (%)	37.3	37.2	37.1	37.3	37.3	37.3
GC content of LSC (%)	35.3	35.2	35.1	35.3	35.3	35.2
GC content of SSC (%)	31.7	31.6	31.4	31.7	31.7	31.6
GC content of IR (%)	43.0	43.0	43.0	43.0	43.0	43.0

### Repeat Sequence Variations

**FIGURE 1:**
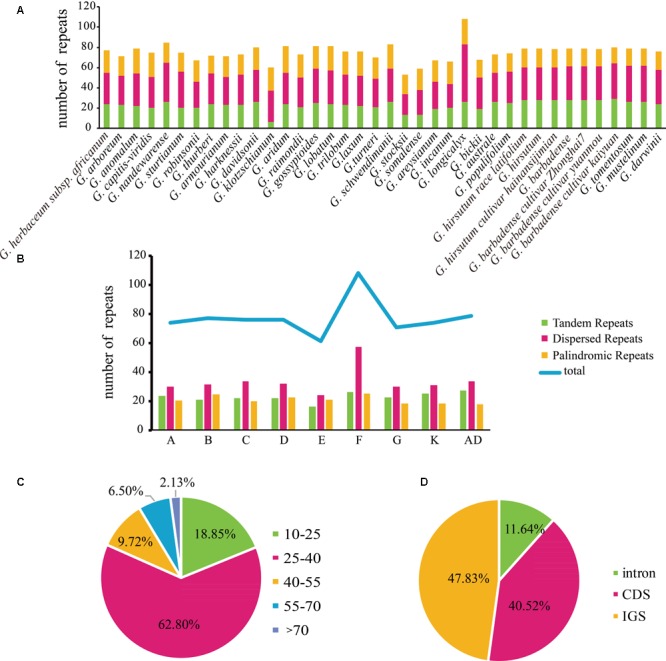
Analysis of repeated sequences in cotton chloroplast genomes. **(A)** Numbers of three repeat types. **(B)** Average number in each genome. **(C)** Summary of repeat sequences by length. **(D)** Distribution of repeat sequences. ^∗^Newly sequenced species in this study.

The chloroplast genomes in the genus *Gossypium* contain numerous tandem repeats, dispersed repeats, and palindromic repeats. In this study, 2860 repeats were identified in cotton plastid genomes, where 1204 comprised dispersed repeats as the most common of the three types, which accounted for 42.10% of the total repeats, as well as 779 palindrome repeats, which accounted for 27.24%, and the number of tandem repeats was 877, which accounted for 30.66% (**Supplementary Figure S2**). The species that contained the most repeated sequences was the F_1_ genome *G. longicalyx*, with 26 tandem repeats, 57 dispersed repeats, and 25 dispersed repeats. The lowest number of repeated sequences was found in the E_1_ genome *G. stocksii*, with 13 tandem repeats, 21 dispersed repeats, and 19 dispersed repeats (**Figure 1A**). In addition, the numbers repeated sequences in the A, B, C, D, G, K, and AD genome groups ranged from 70–80, where the E-genome only contained 61 and F-genome contained 108 (**Figure 1B**). Most of the sequence repeats were located in the non-coding regions, which accounted for 59.47% (intergenic spacers = 47.83% and intron regions = 11.64%) (**Figure 1D**), but some repeats were identified in protein-coding regions, such as *ycf1, ycf2, psaA, psaB, trnS-GCU*, and *trnS-UGA* (**Supplementary Table S5**). Most of the repeats (62.80%) had lengths of 25–40 bp (**Figure 1C** and **Supplementary Table S6**). The numbers of the three sequence repeat types were similar in the cotton plastid genomes and their positions in the chloroplast genomes were relatively conserved.

**FIGURE 2:**
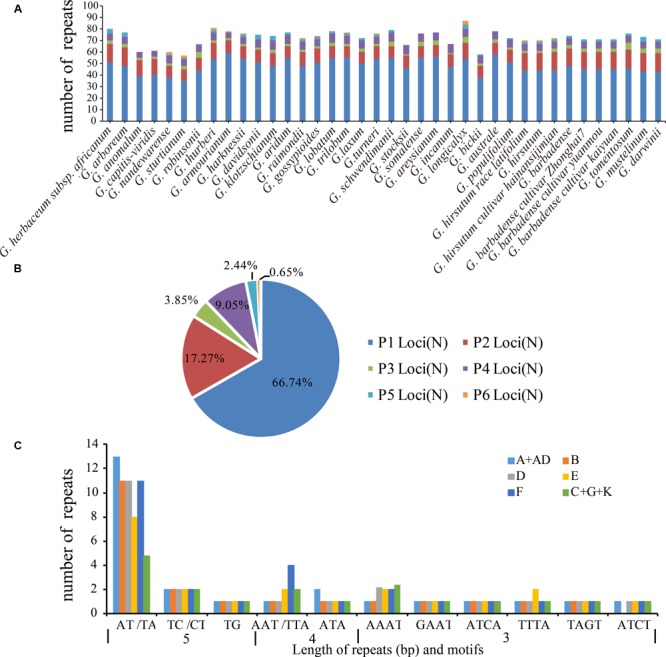
Simple sequence repeats (SSRs) in chloroplast genomes of the genus *Gossypium*. **(A)** Frequency of repeats. **(B)** Summary of SSR loci (P1 to P6 indicate SSR loci with mono-, di-, tri-, tetra-, penta-, and hexanucleotide repeats, respectively). **(C)** Common SSRs types in all six cotton clades.

In addition, SSRs were identified in the cotton plastid genomes (**Figure 2A**). The number of SSRs was highest in the F_1_ genome *G. longicalyx* (87) and that in the C_1_ genome *G. sturtianum* was the lowest (57). In total, 2751 SSRs were identified, where 2101 were found in the LSC region, and 74, 502, and 74 in the IRb, SSC, and IRa regions, respectively. Mononucleotides repeats were most common among these SSRs where they accounted for 66.74% of the total, and dinucleotide repeats accounted for 17.27%. The number of tetranucleotide repeats was slightly highest than that of trinucleotide repeats, but penta- and hexanucleotides were very rare in the cotton plastomes (**Figure 2B**).

In addition, we detected the distributions of SSRs (2–6 repeat units) in the six putative cotton genetic clades (A+AD, F, E, D, B, and C+G+K). Interestingly, dinucleotide repeats (AT/TA) were the most common type in all six cotton clades (**Figure 2C**). There were few penta-and hexanucleotide SSRs in the *Gossypium* species, with three hexanucleotide repeats in the F_1_ genome *G. longicalyx* and two hexanucleotide repeats in seven of the 13 D-genome species (*G. thurberi* D_1_, *G. armourianum* D_2-1_, *G. harknessii* D_2-2_, *G. raimondii* D_5_, *G. gossypioides* D_6_, *G. trilobum* D_8_, and *G. turneri* D_10_). In addition, two C-genome species comprising *G. sturtianum* C_1_ and *G. nandewarense* C_1-n_ contained two hexanucleotide repeats (TTAATA). Among the A+AD genome species, only *G. hirsutum* AD_1_ contained two hexanucleotide repeats (CTTATT). Interestingly, the variations in 23 orthologous SSRs (2–6 repeat units) were shared by all of the cotton plastomes examined in this study, where 18 of these SSR sites were polymorphic and they were mainly located in the intergenic regions of LSCs. The other five loci were monomorphic and they were located in the coding regions of LSCs and SSC (**Supplementary Tables S7, S8**).

### Sequence Divergence

**FIGURE 3:**
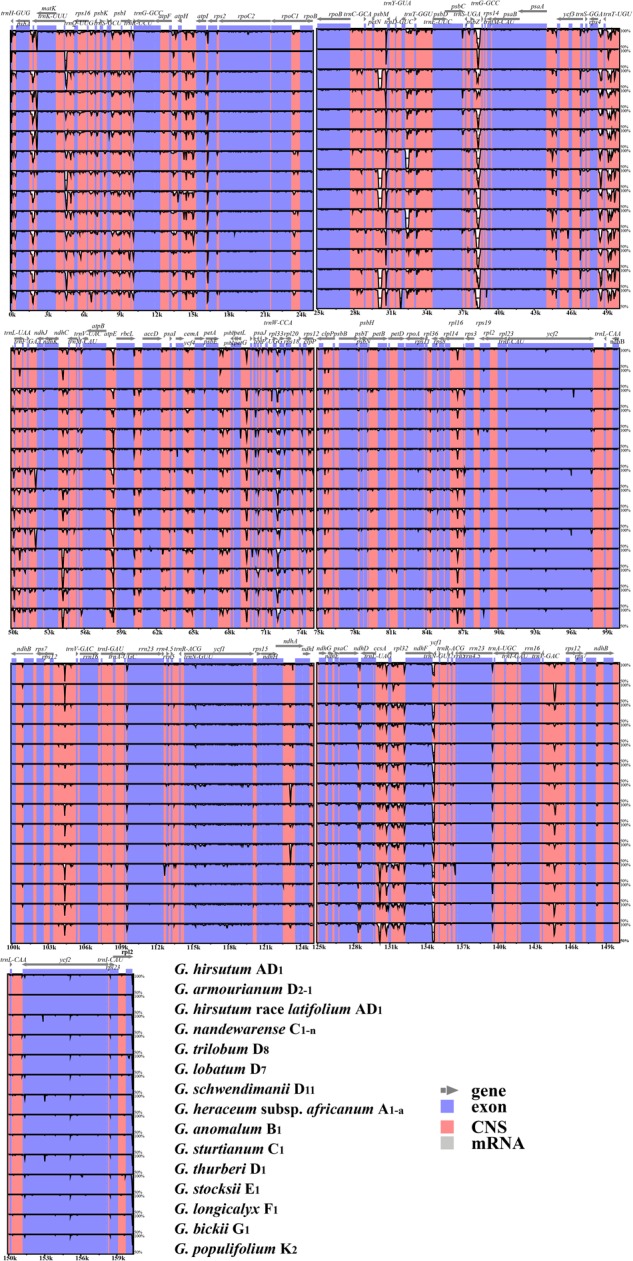
Sequence identity plots based on 15 *Gossypium* chloroplast genomes, with *Gossypium hirsutum* as a reference. Sequences of chloroplast genomes were aligned and compared using the mVISTA program. Annotated genes are displayed along the top. The vertical scale indicates the percentage identity ranging from 50 to 100%. Genome regions are color coded as exon, conserved non-coding sequences (CNS), and mRNA.

Sequence divergence analysis indicated high sequence similarity across the cotton chloroplast genomes (**Figure 3**) and the results suggested that the chloroplast genomes are relatively well conserved. In addition, the non-coding and single copy regions exhibited higher levels of divergence than the IR and coding regions.

**FIGURE 4:**
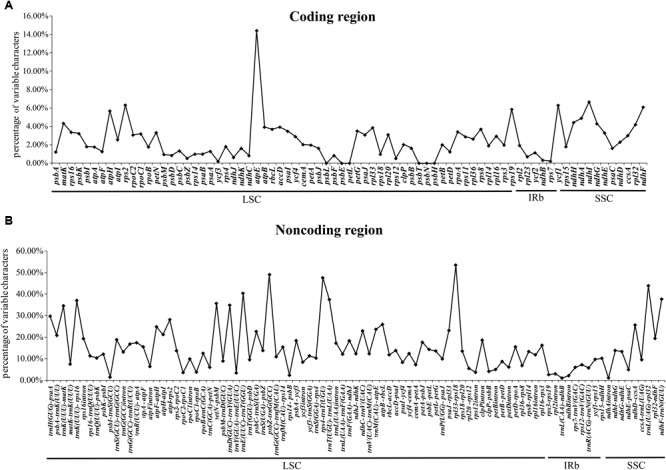
Percentages of variable characters in homologous regions among *Gossypium* chloroplast genomes. **(A)** Coding regions. **(B)** Non-coding regions. The homologous regions are oriented according to their locations in the chloroplast genome.

We also detected divergent hotspot regions in the 40 chloroplast genomes (**Figure 4**). As expected, the sequence divergence in the non-coding regions (including intergenic spacers and introns) ranged from 0.3 to 54.1% with a mean value of 15.1%, which was seven times higher than that in the protein-coding regions (average of 2.5%). Five intergenic regions with percentages exceeding 40% were *rpl33-rps18, psbZ-trnG* (*GCC*), *rps4-trnT* (*UGU*), *trnL* (*UAG*)*-rpl32*, and *trnE* (*UUC*)*-trnT* (*GGU*) (**Figure 4A**). However, the highest proportion of nucleotide variation in genic regions was 14.4%, where five genic regions had percentages exceeding 6%, i.e., *atpE, ndhI, rps2, ycf1*, and *ndhF*, thereby indicating that the coding regions were more highly conserved than the non-coding regions (**Figure 4B**). In addition, the average percentage of variability in IRs (8.00 and 0.87%) was lower than that in the LSC (16.71 and 2.35%) or SSC (18.26 and 4.08%) regions, which demonstrates that the IR regions were highly conserved and they had fewer nucleotide mutations (**Supplementary Table S9**).

### Protein Sequence Evolution

**FIGURE 5:**
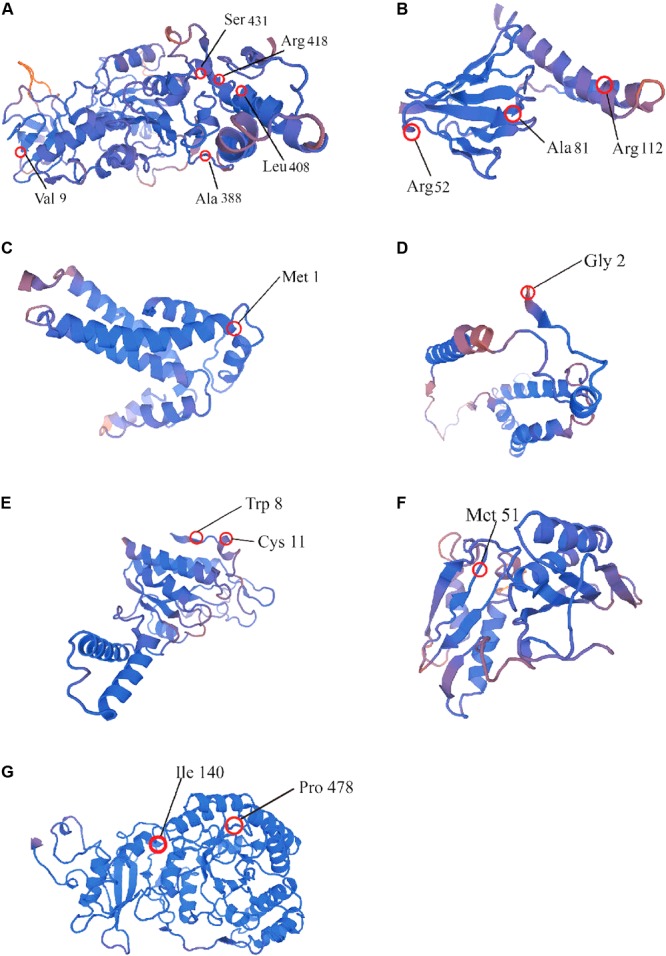
Reference three-dimensional structures of seven positively selected genes: **(A)** *atpB*; **(B)** *atpE*; **(C)** *petB*; **(D)** *petD*; **(E)** *rps2*; **(F)** *rps3*; and **(G)** *rbcL*.

In order to assess the selective pressure on protein-coding genes on cotton plastid genomes, we used the codon substitution models to examine the possible sites under positive selection. The site-specific models allowed ω to vary among sites in all of the cotton phylogenetic branches. We found that 10 coding genes harbored sites under selection (**Supplementary Tables S10, S11**). We found that model 2 and model 8 had better fit to the sequences of these 10 genes based on the LRT and AIC evaluation criteria. These loci included two subunits of the ATP gene (*atpB* and *atpE*), two subunit of ribosome genes (*rps2* and *rps3*), two subunits of cytochrome genes (*petB* and *petD*), and *ccsA, cemA, ycf1*, and *rbcL* genes. Interestingly, four genes had multiple sites under positive selection, i.e., *atpB* (Val 9, Ala 388, Leu 408, Arg 418, and Ser 431), *atpE* (Arg 52, Ala 81, and Arg 112), *ycf1* (Val 49, Ser 90, Met 407, Ser 970, Leu 1004, and Asn 1196), and *rps2* (Trp 8 and Cys 11), whereas the other six genes had only one site under positive selection. The reference three-dimensional structures of the *atpB, atpE, rps2, rps3, petB, petD*, and *rbcL* genes were constructed and the sites under positive selection are marked in **Figure 5**.

### Phylogenetic Analysis

**FIGURE 6:**
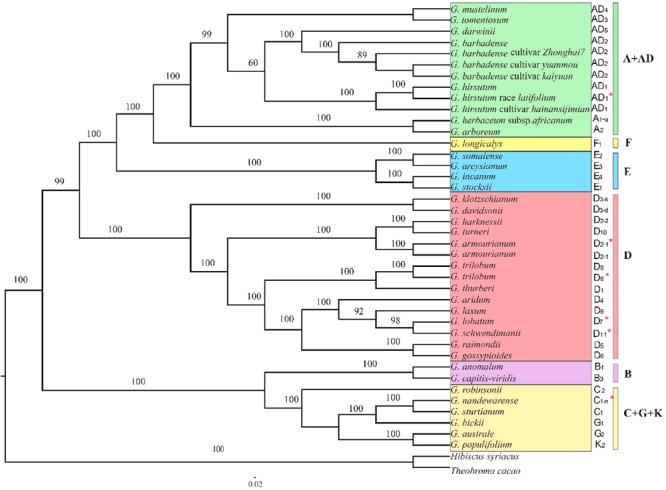
Phylogenetic relationships among the 40 *Gossypium* species based on complete chloroplast genome sequences. Different colors represent six genetic clades and red asterisks indicate the newly sequenced species. *Hibiscus syriacus* and *Theobroma cacao* were used as outgroups.

Maximum likelihood phylogenetic analysis using two different data sets (all of the chloroplast genomes and 78 concatenated plastid protein-coding genes) generated identical topologies for the 40 cotton species examined this study (**Figure 6**). The analysis obtained moderate to high support for nearly all of the nodes. The six major genetic clades were identified, i.e., the A+AD, F, E, D, B, and C+G+K genome groups. As expected, all 13 D-genome species formed a strong monophyletic genetic clade, whereas the newly sequenced *G*. *armourianum* (D_2-1_) and previously published plastomes of the same species KP221926 clustered into a small clade, in a similar manner to the newly released *G. trilobum* plastome and that published previously for KP170503. In addition, *G. harknessii* (D_2-2_) appeared to be more closely related to *G. turneri* (D_10_) than *G. armourianum* (D_2-1_). The newly sequenced *G. lobatum* (D_7_) and *G. schwendimanii* (D_11_) formed a small monophyletic clade, and they were closely related to *G. laxum* (D_9_) and *G. aridum* (D_4_) in a larger branch. It was notable that in the Australian basal clade (C+G+K), *G. nandewarense* (C_1-n_) and *G. sturtianum* (C_1_) formed a small monophyletic clade, but they were separated from *G. robinsonii* (C_2_) instead of being closed allied with *G. bickii* (G_1_). In addition, the semi-wild race of upland cotton *G. hirsutum* race *latifolium* (AD_1_) was closely related to *G. hirsutum* (AD_1_). The 10 allotetraploid cotton plastomes were closely clustered together, and the A-genome was closest to the tetraploid branch.

## Discussion

### Chloroplast Sequence Variation

The genome size, gene order, and compositions in the 40 cotton chloroplast genomes examined in this study were similar to those found in previously sequenced *Gossypium* plastid genomes, where they ranged in size from 159 to 161 kb (Chen et al., 2017a). All of the newly examined chloroplast genomes of *Gossypium* species contained more AT and they had GC contents of about 37.1–37.4%, which are similar to those in most land plants (Bock, 2007). The GC contents of the chloroplast genomes were much lower in the non-coding intergenic regions than the coding regions. Interestingly, the GC contents of the IR regions were higher than those of the other plastid DNA regions, possibly due to the presence of rRNA genes (Bock, 2007). Interestingly, we determined that the differences in the GC content were smaller in the IR regions than those in the LSC and SSC regions in the different cotton species, thereby suggesting that conservation of the IR regions was possibly correlated with their higher GC contents.

In addition, the *Gossypium* plastomes contained a high frequency of large repeats. Previous studies have suggested that larger and complex repeat sequences have played key roles in sequence rearrangements and chloroplast genome evolution (Milligan et al., 1989; Cavalier-Smith, 2002; Bausher et al., 2006; Huang et al., 2014). In this study, the F_1_ genome *G. longicalyx* had the highest numbers of repeats (108), whereas the E_1_ genome *G. stocksii* had the lowest (53). We replicated the analyses of the repeats in the different cotton species, where the techniques and methods used to sequence and assemble the different genomes were comparable. Interestingly, we identified a weak significant correlation (*R*^2^ = 0.265, *P* < 0.05) between the number of repeats and the size of the plastid genomes in the cotton species (Supplementary Figure S3), although this could possibly have been due to differences caused by artifacts in the repeats. The large amount of repeat sequences extends our knowledge of chloroplast genomes in cotton species. A similar pattern was also identified in algal plastomes (Maul et al., 2002; Pombert et al., 2005).

From an evolutionary viewpoint, variations in repeated sequence among species are due to natural selection and adaptation by organisms to diverse environments (Britten and Kohne, 1968), where the proportion of repetitive sequences relative to the total amount of DNA is greater with a higher level of biological evolution (Britten and Davidson, 1971). In general, from prokaryotes to eukaryotes, the increasing number of repetitive sequences in the genome protects the coding sequences and facilitates evolution to generate new genes that are a necessary consequence of evolution (Britten and Davidson, 1971; Elder and Turner, 1995; Cosner et al., 1997). In this study, dispersed repeats were the most abundant type of repeat in the *Gossypium* chloroplast genomes, as also reported in other angiosperm lineages such as *Trachelium* (Haberle et al., 2008). The number of repeats in the same genome was almost similar, but there were large differences in the number of repeats in various genomes. In addition, the distributions of tandem, dispersed, and palindromic repeats were highly similar in all of the *Gossypium* species, and they were usually located in the same genes (psaA, psaB, ycf1, ycf2, trnS-GCU, and trnS-UGA). This large amount of repeats might maintain the stability of chloroplast genomes, and similar results were also obtained in other studies (Maréchal and Brisson, 2010).

In addition, the SSRs in the cotton chloroplast genomes contained many AT units, where all of the mononucleotide SSRs comprised A/T repeats. This result is largely consistent with previous reports that chloroplast SSRs are dominated by A/T repeats and that they contribute greatly to the AT richness of plastid genomes (Nie et al., 2012). In addition, mononucleotide repeats were common and they accounted for 66.54% of the total SSRs. It is well known that long mononucleotide stretches are perfect hotspots for sequencing errors, and thus they are always considered highly error-prone. Therefore, we determined the distributions of SSRs (2–6 repeat units) in the genetic clades of cotton comprising A+AD, F, E, D, B, and C+G+K. Interestingly, dinucleotide repeats (AT/TA) were the most common of the different types in all six cotton clades, whereas few penta- and hexanucleotide SSRs occurred in the genus *Gossypium*. In general, the variability in the copy number of SSRs is highly polymorphic in the chloroplast genome and these variations can be used as molecular genetic markers in studies of population genetics, phylogeography (Xue et al., 2012), phylogeny, and species identification (Stanford et al., 2000; Aradhya et al., 2007; Wang et al., 2013). In this study, 23 orthologous SSR variations were shared by all of the cotton plastomes (the most common type comprised dinucleotide repeats, followed by tetra- and pentanucleotides). Among these SSR variations, 18 were polymorphic and they were mainly located in the intergenic regions of LSCs. These polymorphic sites could be useful molecular markers in further studies of population genetics and phylogeography.

In addition, among all 40 *Gossypium* chloroplast genome sequences, the nucleotide variations were more conserved in the IR regions than the SC regions, and similar results have been obtained in most angiosperms (Khakhlova and Bock, 2006; Zhang et al., 2016). Ten divergence hotspots [*rpl33-rps18, psbZ-trnG (GCC), rps4-trnT (UGU), trnL (UAG)-rpl32, trnE (UUC)-trnT (GGU), atpE, ndhI, rps2, ycf1*, and *ndhF*] were identified in *Gossypium* chloroplast genome sequences, which could be used to develop universal primers and candidate DNA barcodes in the future.

### Protein Sequence Evolution

Variations in synonymous and non-synonymous nucleotide sites are very useful molecular markers for studies of evolutionary biology. In this study, we detected 10 chloroplast protein-coding genes that exhibited site-specific selection. Interestingly, *atpB, atpE, rps2*, and *ycf1* were found to harbor five, three, two, and five sites under selection, respectively. The *atpE* gene is co-transcribed and coupled with *atpB* (Chotewutmontri and Barkan, 2016), and it plays a key role in the plant development process (Chotewutmontri and Barkan, 2016). The high degree of variability in *atpE* might indicate the potential for positive selection to fine tune the demand for the rapid activation of the ATP (Rott et al., 2011). In addition, we detected positively selected sites in the *rbcL* gene, which is essential as a modulator of photosynthetic (Allahverdiyeva et al., 2005). A previous analysis of the evolution of 113 species of PACMAD grasses (Poaceae) by Piot et al. (2018) demonstrated that *rbcL* underwent strong positive selection during the C3–C4 photosynthetic transitions. In addition, we observed site-specific selection in *rps2* and *rps3* genes, which have important roles in the chloroplast ribosome (Rogalski et al., 2006, 2008; Tiller and Bock, 2014). Moreover, the *petB* and *petD* genes are crucial for the synthesis of the cytochrome b_6_/f (Cyt b_6_/f) complex, which affects linear and cyclic electron transport functions (Xiao et al., 2012), and they are under significant adaptive selection. These positively selected genes may have played important roles in the adaptation of cotton species to diverse environments.

### Phylogenetic Relationships

Many studies have determined the molecular phylogenetic relationships of cotton species based on limited numbers of plastid and nuclear DNA markers, as well as entire chloroplast and mitochondrial genome data sets (Cronn et al., 2002; Senchina et al., 2003; Wendel et al., 2009; Xu et al., 2012; Wendel and Grover, 2015; Chen et al., 2016, 2017a,b). The results obtained in previous studies indicate that the main evolutionary branches of cotton species can be well resolved, but the topologies of chloroplast and nuclear gene markers sometimes differ in terms of their species relationships in some clades in the cotton genus (Cronn et al., 2002; Chen et al., 2016, 2017a). In order to explore this inconsistency in depth, we conducted phylogenetic analyses based on 40 *Gossypium* plastid genome sequences, which represented the greatest diversity known in the cotton genus, and the structure of the tree obtained was mainly consistent with previous analyses. In the phylogenetic tree, the *Gossypium* species were primarily divided into two large genetic branches, where one included all of the Australian species with C, G, and K genomes, and the other included all of the American species, the African-Asian species, the B genome located in the clade of Australian species, and the A, E, and F genomes in the clade of American species. Furthermore, all of the species were grouped into six major cotton genetic clades, i.e., A+AD, F, E, D, B, and C+G+K, which is largely consistent with previously reported results based on the 78 concatenated chloroplast protein-coding genes (Chen et al., 2016, 2017a). In addition, phylogenetic tree analysis identified very close relationships between the A and AD genome species. The results confirmed that the maternal donor of allotetraploid species probably belonged to A genome species (Cronn et al., 2002; Chen et al., 2016, 2017a). In the A+AD group, the newly sequenced *G. hirsutum* race *latifolium* and *G. hirsutum* formed an individual monophyletic branch with a high bootstrap support value.

Moreover, the newly reported *G. nandewarense* (C_1-n_) and *G. sturtianum* (C_1_) were closely related in the ML tree, where they clustered in an individual genetic clade together with the G-genome *G. bickii* (G_1_). It was notable that the G-genome *G. australe* (G_2_) and K-genome *G. populifolium* grouped into a monophyletic clade. Our results are largely consistent with previous phylogenetic analyses based on the 78 protein-coding genes but using samples of different cotton species (Chen et al., 2017a). In the present study, we did not consider the previously released data sets for two species comprising *G. nelsonii* (G-genome) and *G. pilosum* (K-genome) reported by Chen et al. (2017a) due to inconsistencies between the data records in their study and GenBank, and the chloroplast genomes were only partially sequenced for *G. pilosum*. However, the major phylogenetic framework for C+G+K genome species was basically consistent with that found in previous studies (Chen et al., 2016, 2017a). The species from the C, G, and K genomes were largely admixed and nested in a larger evolutionary branch. As shown in some other studies, the G-genome species *G. bickii* had common nested relationships with the C-genome species, possibly due to frequent chloroplast capture in the *G. bickii* lineage (Seelanan et al., 1999; Liu et al., 2001). In addition, the two G-genome species *G. bickii* and *G. australe* clustered into two different genetic clades, which possibly reflected their recent speciation and/or ancient hybridization events among the different cotton species. Recent rapid species radiation could have resulted in incomplete lineage sorting among closely related species, thereby explaining the inconsistent results obtained based on different inherited property DNA markers (Seelanan et al., 1999; Liu et al., 2001). Interestingly, some previous studies also suggested higher levels of introgression hybridization and radiation divergence in these cotton species (Cronn and Wendel, 2004; Li et al., 2014).

In the present study, the addition of four newly reported D-genome cotton species to the nine previously released D-genome chloroplasts (Xu et al., 2012; Chen et al., 2016, 2017a) allowed us to conduct the first widespread phylogenetic reconstruction of the D-genome using the whole plastid genome sequences. The phylogenetic tree showed that all 13 species clustered into a strong monophyletic clade. Some species–pair relationships were identified, including *G. davidsonii* (D_3-d_) with *G. klotzschianum* (D_3-k_), *G. harknessii* (D_2-2_) with *G. turneri* (D_10_), *G. trilobum* (D_8_) with *G. thurberi* (D_1_), and *G. raimondii* (D_5_) with *G. gossypioides* (D_6_), which were largely consistent with previous reports based on limited nuclear loci, SSR-based DNA markers, and whole plastomes (Álvarez et al., 2005; Ulloa et al., 2013; Chen et al., 2017a). Interestingly, as reported previously for low-copy nuclear genes (Cronn and Wendel, 2004), the three Mexican arborescent cotton species comprising *G. laxum, G. lobatum*, and *G. schwendimanii* were sequenced for the first time and they formed a small monophyletic clade with high bootstrap support, where the latter two species had a close relationship. *G. aridum* was nested in the outside branch of the three arborescent cotton species. We also detected high levels of congruent and/or incongruent results with respect to the relationships of the two D-genome species: *G. raimondii* (D_5_) and *G. gossypioides* (D_6_). The present study based on whole plastomes showed that the two species formed a strong monophyletic clade, which is consistent with previous reports based on a few chloroplast DNA regions and whole plastomes sampled from a limited number of D-genome species (Cronn et al., 2002; Chen et al., 2016, 2017a). However, largely inconsistent results were obtained using biparentally inherited nuclear markers (Cronn and Wendel, 2004). These discrepancies in the phylogenetic relationships may be explained by the different inherited properties of the DNA markers employed and the network of connections within the cotton genus, which requires further exploration. In conclusion, the phylogenetic analyses conducted in this study based on whole chloroplast genome sequences provide the basis for resolving the relationships among the major clades of *Gossypium* species.

## Author Contributions

Z-HL designed and conceived the study. YW, FL, and D-GY performed the experiments. FL, WL, X-JZ, X-YP, Y-GL, K-LH, W-SZ, and Z-YR contributed the materials/analysis tools. Z-HL, X-FM, and YW wrote the manuscript. Z-HL, X-FM, YW, and K-HZ revised the manuscript.

## Conflict of Interest Statement

The authors declare that the research was conducted in the absence of any commercial or financial relationships that could be construed as a potential conflict of interest.

## Footnotes

1 https://swissmodel.expasy.org/

## Supplementary Material

FIGURE S1 Chloroplast genome maps of six *Gossypium* species. The genes shown outside the circle are transcribed clockwise, whereas those inside are transcribed counterclockwise. Genes belonging to different functional groups are color coded. The dashed gray area in the inner circle shows the proportional GC content of the corresponding genes. LSC, SSC, and IR denote large single copy, small single copy, and inverted repeat regions, respectively.

FIGURE S2 Pie graph representations of the three types of repeats.

FIGURE S3 Relationships between *Gossypium* chloroplast genome sizes and the number of repeats.

TABLE S1 Sampling and assembly information, and accession numbers for six *Gossypium* species.

TABLE S2 Primers for small gaps and ambiguous sequences.

TABLE S3 General features of 38 *Gossypium* species and the outgroup chloroplast genomes.

TABLE S4 List of genes present in six *Gossypium* chloroplast genomes.

TABLE S5 Lengths of dispersed repeats and distribution of palindromic repeats in 38 *Gossypium* chloroplast genomes.

TABLE S6 Distribution of tandem repeat lengths in cotton species.

TABLE S7 Distribution of SSRs in cotton chloroplast genomes.

TABLE S8 Orthologous SSR (2–6 repeat units) variations across all the cotton plastomes examined in this study.

TABLE S9 Percentages of variable characters in coding and non-coding regions of 38 *Gossypium* chloroplast genomes.

TABLE S10 Maximum likelihood parameter estimates for the positively selected genes and the AIC values under different models.

TABLE S11 Likelihood ratio test (LRT) of the variable ω ratio under different models.
