# Supplementary figures and images for: Comparative Chloroplast Genomics of Gossypium Species: Insights Into Repeat Sequence Variations and Phylogeny
Source: Front Plant Sci. 2018 Mar 21;9:376. doi: 10.3389/fpls.2018.00376 (PMC5871733; doi:10.3389/fpls.2018.00376)

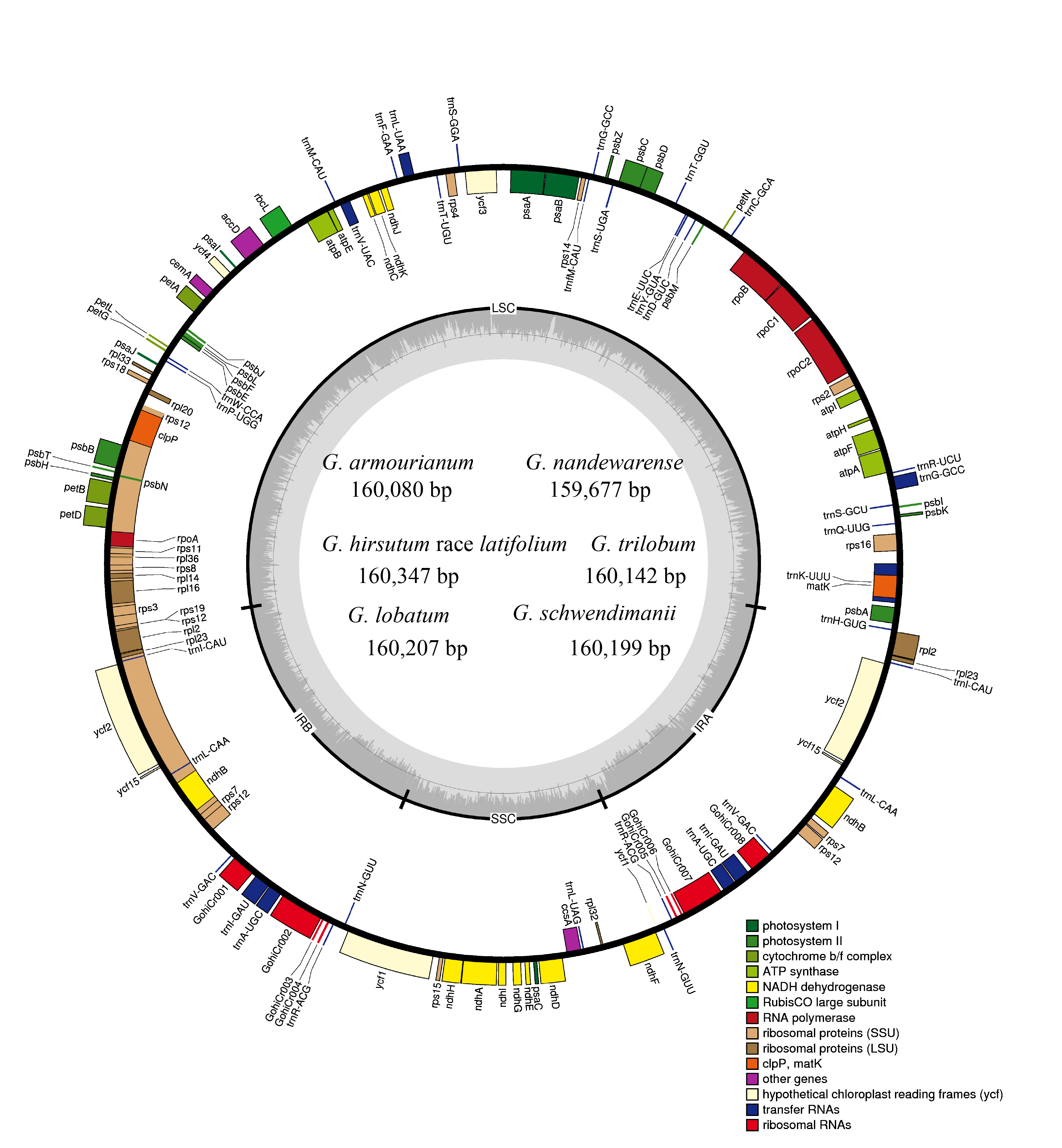

Supplement: FIGURE S1 — Chloroplast genome maps of six Gossypium species. The genes shown outside the circle are transcribed clockwise, whereas those inside are transcribed counterclockwise. Genes belonging to different functional groups are color coded. The dashed gray area in the inner circle shows the proportional GC content of the corresponding genes. LSC, SSC, and IR denote large single copy, small single copy, and inverted repeat regions, respectively. [file Image_1.TIF]

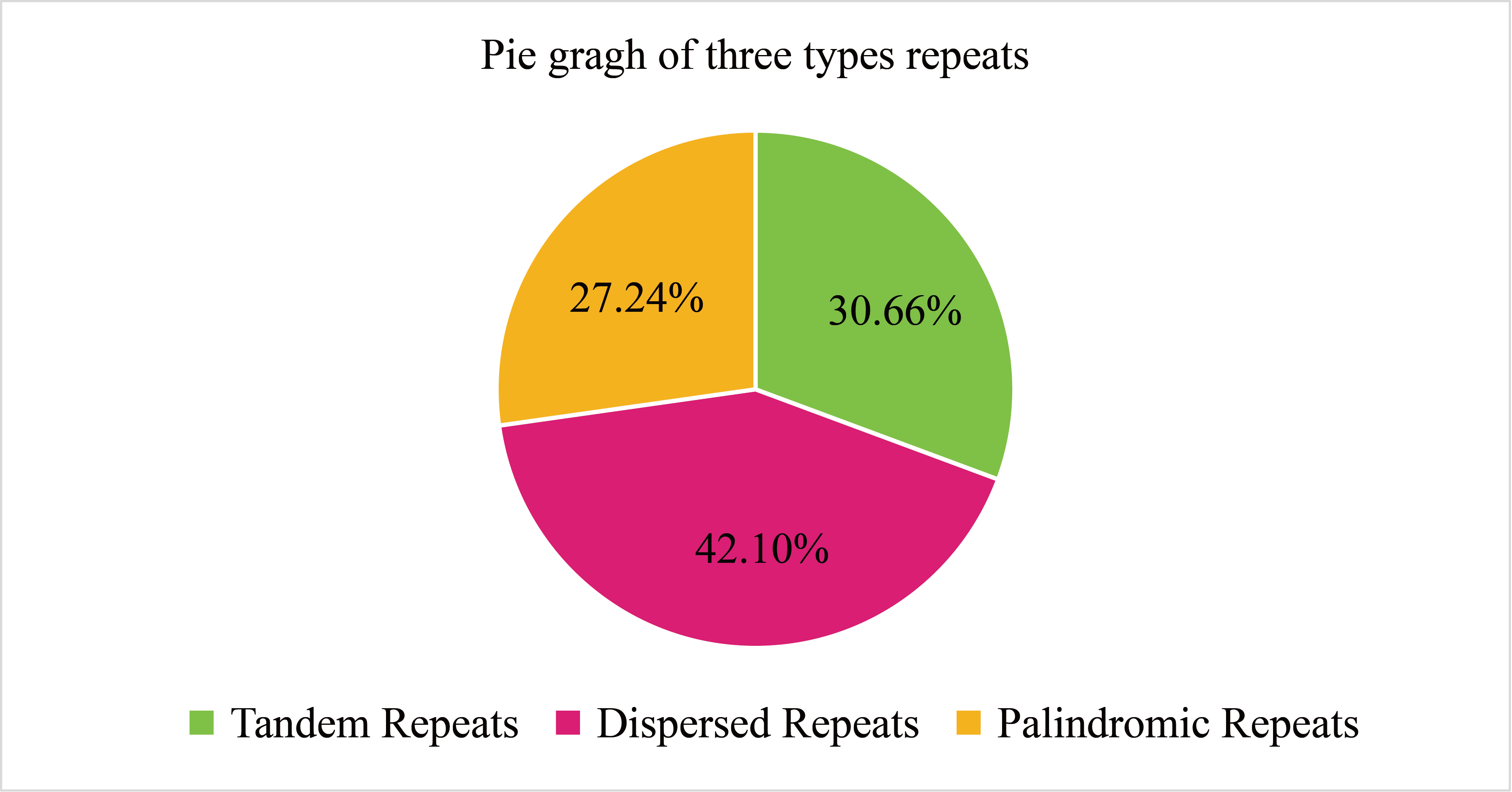

Supplement: FIGURE S2 — Pie graph representations of the three types of repeats. [file Image_2.TIF]

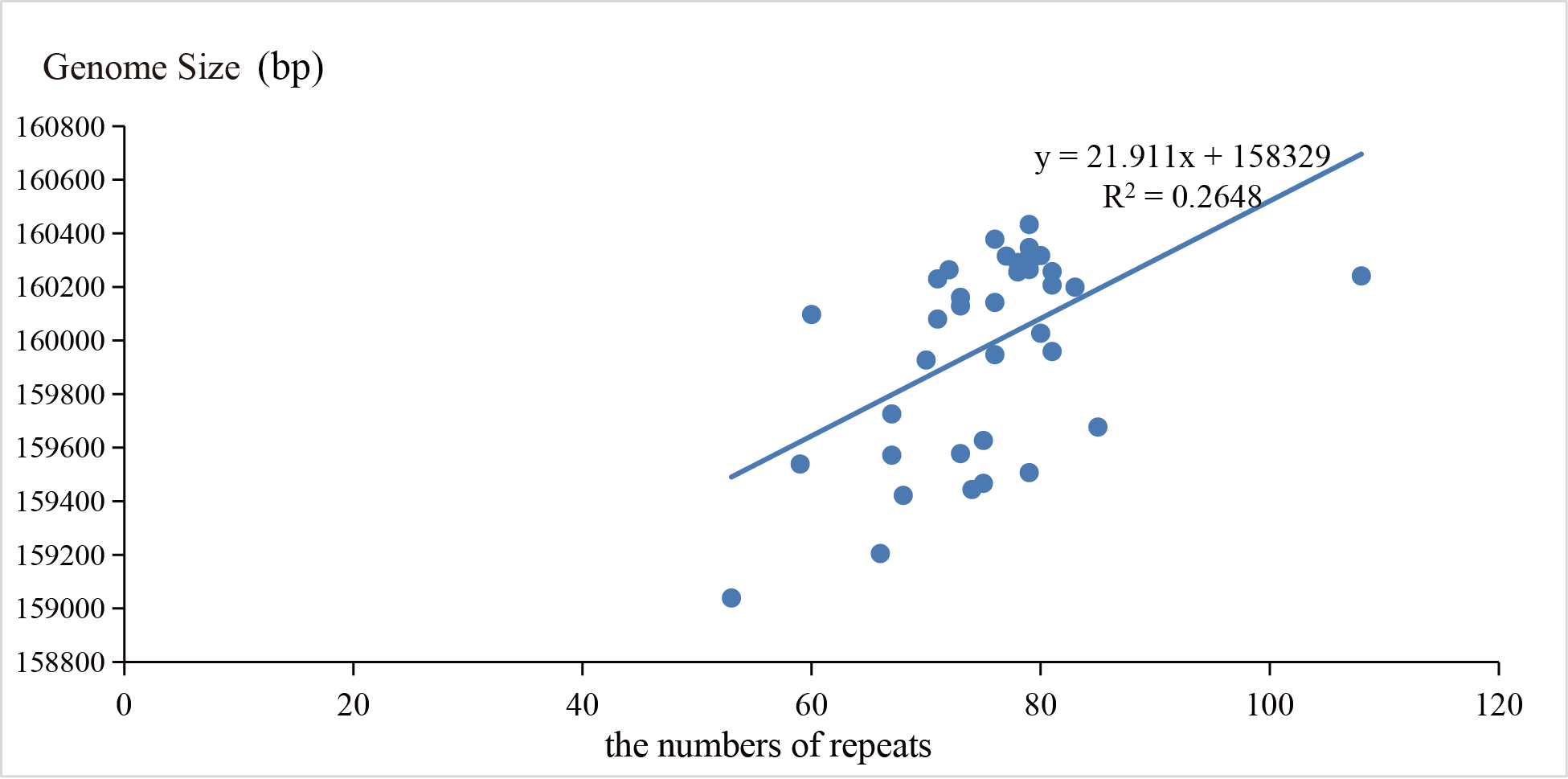

Supplement: FIGURE S3 — Relationships between Gossypium chloroplast genome sizes and the number of repeats. [file Image_3.TIF]
