# Supplementary material for: Comparative Chloroplast Genomics of Gossypium Species: Insights Into Repeat Sequence Variations and Phylogeny
Source: Front Plant Sci. 2018 Mar 21;9:376. doi: 10.3389/fpls.2018.00376 (PMC5871733; doi:10.3389/fpls.2018.00376)
Supplement: TABLE S1 — Sampling and assembly information, and accession numbers for six Gossypium species. [file Table_1.DOCX]

**Table S1** Sampling and assembly information, and accession numbers for six *Gossypium* species.

| Species | Locality | Clean reads | Assembly reads | Mean length of reads | Mean coverage | Accession number in GenBank |
| --- | --- | --- | --- | --- | --- | --- |
| *G. armourianum* | Sanya, Hainan,China | 7,378,504 | 1,195,636 | 150.1 | 1113.7 | MG891801 |
| *G.hirsutum* race*latifolium* | Sanya, Hainan,China | 6,560,002 | 598.937 | 150.3 | 559.5 | MG800784 |
| *G.nandewarense* | Sanya, Hainan,China | 11,071,396 | 1,006,955 | 150.2 | 937.3 | MG779276 |
| *G.trilobum* | Sanya, Hainan,China | 4,988,282 | 1,120,261 | 150.1 | 1032.7 | MG800783 |
| *G. lobatum* | Sanya, Hainan,China | 4,455.952 | 649,184 | 150.2 | 606.6 | MG891802 |
| *G. schwendimanii* | Sanya, Hainan,China | 4,085,572 | 214,875 | 150.7 | 201.9 | MG891803 |
