# Supplementary material for: Comparative Chloroplast Genomics of Gossypium Species: Insights Into Repeat Sequence Variations and Phylogeny
Source: Front Plant Sci. 2018 Mar 21;9:376. doi: 10.3389/fpls.2018.00376 (PMC5871733; doi:10.3389/fpls.2018.00376)
Supplement: TABLE S2 — Primers for small gaps and ambiguous sequences. [file Table_2.DOC]

**Table S2.** Primers for small gaps and ambiguous sequences.

| Species | Region amplified |  | Primer sequence | Tm |
| --- | --- | --- | --- | --- |
| *G. trilobum* | LSC; IGS; *trnE-UUC-trnT-GGU* | F | CCTGACCGCGACCATACTAT | 57.6 |
| *G. trilobum* | LSC; IGS; *trnE-UUC-trnT-GGU* | R | ACATGCCTGTCTCGTCGTAA | 55.4 |
| *G. trilobum* | SSC; IGS; *ndhF-trnN-GUU* | F | GGTATAAGAGGATTGGCAGAACT | 56 |
| *G. trilobum* | SSC; IGS;*ndhF-trnN-GUU* | R | CCATCCAGATCCCAATTCCATTT | 56 |
| *G. armourianum* | LSC; *rpl16 intron* | F | GCTCTCGATTAATGCTTAGG | 53.4 |
| *G. armourianum* | LSC; *rpl16 intron* | R | TAGATCGAAATTGTGAAGCA | 49.3 |
| *G. armourianum* | SSC; IGS; *ndhF-trnN-GUU* | F | GGTATAAGAGGATTGGCAGAACT | 56 |
| *G. armourianum* | SSC; IGS; *ndhF-trnN-GUU* | R | CCATCCAGATCCCAATTCCATTT | 56 |

Note: F and R represent “Forward” and “Reverse,” respectively.
