# Supplementary material for: Comparative Chloroplast Genomics of Gossypium Species: Insights Into Repeat Sequence Variations and Phylogeny
Source: Front Plant Sci. 2018 Mar 21;9:376. doi: 10.3389/fpls.2018.00376 (PMC5871733; doi:10.3389/fpls.2018.00376)
Supplement: TABLE S3 — General features of 38 Gossypium species and the outgroup chloroplast genomes. [file Table_3.DOCX]

| **Table S3** General features of 38 *Gossypium* species and the outgroup chloroplast genomes. | | | | | | | | | | |
| --- | --- | --- | --- | --- | --- | --- | --- | --- | --- | --- |
| species | Sub-groups | SSC (bp) | GC content  (%) | IR (bp) | GC content  (%) | LSC (bp) | GC content  (%) | Length (bp) | Overall GC content (%) | Accession No. |
| *G. herbaceum subsp.africanum* | A1-a | 20285 | 31.6 | 25620 | 42.90 | 88790 | 35.2 | 160315 | 37.2 | NC_016692 |
| *G. arboreum* | A_2_ | 20274 | 31.6 | 25617 | 43.0 | 88722 | 35.2 | 160230 | 37.2 | NC_016712 |
| *G. anomalum* | B_1_ | 20199 | 31.6 | 25602 | 43.0 | 88102 | 35.3 | 159507 | 37.3 | NC_023213 |
| *G. capitis-viridis* | B_3_ | 20198 | 31.6 | 25602 | 43.0 | 88065 | 35.3 | 159467 | 37.3 | NC_018111 |
| *G. sturtianum* | C_1_ | 20216 | 31.4 | 25580 | 43.0 | 88251 | 35.1 | 159627 | 37.1 | NC_023218 |
| *G. robinsonii* | C_2_ | 20203 | 31.4 | 25582 | 43.0 | 88359 | 35.1 | 159726 | 37.2 | NC_018113 |
| *G. nandewarense* | C_1-n_ | 20241 | 31.4 | 25576 | 43.0 | 88284 | 35.1 | 159677 | 37.1 | MG779276 |
| *G. thurberi* | D_1_ | 20271 | 31.7 | 25628 | 43.0 | 88737 | 35.3 | 160264 | 37.3 | NC_015204 |
| *G. harknessii* | D_2-2_ | 20221 | 31.7 | 25599 | 43.0 | 88710 | 35.3 | 160129 | 37.3 | NC_033333 |
| *G. davidsonii* | D_3-d_ | 20240 | 31.7 | 25602 | 43.0 | 88628 | 35.3 | 160027 | 37.3 | NC_033395 |
| *G. klotzschianum* | D_3-k_ | 20235 | 31.8 | 25604 | 43.0 | 88654 | 35.3 | 160097 | 37.3 | NC_033394 |
| *G. aridum* | D_4_ | 20243 | 31.7 | 25647 | 43.0 | 88720 | 35.2 | 160257 | 37.3 | NC_033396 |
| *G. raimondii* | D_5_ | 20205 | 31.7 | 25650 | 43.0 | 88654 | 35.3 | 160161 | 37.3 | NC_016668 |
| *G. gossypioides* | D_6_ | 20004 | 31.8 | 25576 | 43.0 | 88803 | 35.3 | 159959 | 37.3 | NC_017894 |
| *G. armourianum* | D_2-1_ | 20241 | 31.7 | 25591 | 43.0 | 88657 | 35.3 | 160080 | 37.3 | MG891801 |
| *G. lobatum* | D_7_ | 20294 | 31.7 | 25550 | 43.0 | 88811 | 35.3 | 160205 | 37.3 | MG891802 |
| *G. trilobum* | D_8_ | 20233 | 31.7 | 25587 | 43.0 | 88735 | 35.3 | 160142 | 37.3 | MG800783 |
| *G. schwendimanii* | D_11_ | 20318 | 31.6 | 25551 | 43.0 | 88779 | 35.2 | 160199 | 37.3 | MG891803 |
| *G.laxum* | D_9_ | 20240 | 31.7 | 25683 | 43.0 | 88582 | 35.2 | 159947 | 37.3 | KF806549 |
| *G.turneri* | D_10_ | 20215 | 31.7 | 25650 | 42.9 | 88412 | 35.2 | 159927 | 37.3 | NC_026835 |
| *G. stocksii* | E_1_ | 20179 | 31.7 | 25487 | 43.0 | 87886 | 35.4 | 159039 | 37.4 | NC_023217 |
| *G. somalense* | E_2_ | 20251 | 31.7 | 25569 | 43.0 | 88150 | 35.4 | 159539 | 37.4 | NC_018110 |
| *G. areysianum* | E_3_ | 20252 | 31.7 | 25569 | 43.0 | 88182 | 35.4 | 159572 | 37.4 | NC_018112 |
| *G. incanum* | E_4_ | 20196 | 31.7 | 25565 | 43.0 | 87879 | 35.4 | 159205 | 37.4 | NC_018109 |
| *G. longicalyx* | F_1_ | 20278 | 31.6 | 25648 | 42.9 | 88667 | 35.2 | 160241 | 37.2 | NC_023216 |
| *G. bickii* | G_1_ | 20183 | 31.5 | 25583 | 43.0 | 88073 | 35.2 | 159422 | 37.2 | NC_023214 |
| *G. australe* | G_2_ | 20221 | 31.4 | 25567 | 43.0 | 88223 | 35.1 | 159578 | 37.2 | NC_033401 |
| *G. populifolium* | K_2_ | 20093 | 31.5 | 25577 | 43.0 | 88197 | 35.1 | 159444 | 37.2 | NC_033398 |
| *G. hirsutum* race *latifolium* | AD_1_ | 20287 | 31.6 | 25606 | 43.0 | 88848 | 35.2 | 160347 | 37.2 | MG800784 |
| *G. hirsutum*cultivar*hainansijimian* | AD_1_ | 20279 | 31.6 | 25602 | 43.0 | 88782 | 35.2 | 160265 | 37.2 | HQ901197 |
| *G. hirsutum* | AD_1_ | 20269 | 31.6 | 25608 | 42.9 | 88816 | 35.2 | 160301 | 37.2 | NC_007944 |
| *G. barbadense* | AD_2_ | 20294 | 31.7 | 25591 | 43.0 | 88841 | 35.2 | 160317 | 37.2 | NC_008641 |
| *G. barbadense* cultivar *Zhonghai7* | AD_2_ | 20267 | 31.7 | 25593 | 43.0 | 88849 | 35.2 | 160302 | 37.2 | HQ901199 |
| *G. barbadense* cultivar *Yuanmou* | AD_2_ | 20267 | 31.7 | 25593 | 43.0 | 88838 | 35.2 | 160291 | 37.2 | HQ901198 |
| *G. barbadense*cultivar*kaiyuan* | AD_2_ | 20267 | 31.7 | 25594 | 43.0 | 88836 | 35.2 | 160291 | 37.2 | HQ901200 |
| *G. tomentosum* | AD_3_ | 20271 | 31.7 | 25615 | 42.9 | 88932 | 35.2 | 160433 | 37.2 | NC_016690 |
| *G. mustelinum* | AD_4_ | 20269 | 31.7 | 25609 | 42.9 | 88826 | 35.2 | 160313 | 37.2 | NC_016711 |
| *G. darwinii* | AD_5_ | 20266 | 31.7 | 25603 | 43.0 | 88906 | 35.2 | 160378 | 37.2 | NC_016670 |
| *Theobroma cacao* |  | 20194 | 32.1 | 25546 | 42.9 | 89333 | 34.7 | 160619 | 36.9 | NC_014676 |
| *Hibiscus syriacus* |  | 19831 | 31.1 | 25745 | 42.8 | 89698 | 34.7 | 161019 | 36.8 | NC_026909 |
