# Supplementary material for: Comparative Chloroplast Genomics of Gossypium Species: Insights Into Repeat Sequence Variations and Phylogeny
Source: Front Plant Sci. 2018 Mar 21;9:376. doi: 10.3389/fpls.2018.00376 (PMC5871733; doi:10.3389/fpls.2018.00376)
Supplement: TABLE S4 — List of genes present in six Gossypium chloroplast genomes. [file Table_4.DOCX]

**Table S4.** List of genes present in six *Gossypium* chloroplast genomes.

| **Category** | **Gene group** | **Gene name** | | | | |
| --- | --- | --- | --- | --- | --- | --- |
| Self-replication | Ribosomal RNA | *rrn4.5^a^* | *rrn5^a^* | *rrn16^a^* | *rrn23^a^* |  |
|  | genes |  |  |  |  |  |
|  | Transfer RNA | *trnA-UGC^ab^*  *trnfM-CAU*  *trnI-CAU^a^*  *trnM-CAU*  *trnR-UCU*  *trnT-UGU* | *trnC-GCA*  *trnG-GCC^b^*  *trnK-UUU^b^*  *trnN-GUU^a^*  *trnS-GCU*  *trnV-GAC*^a^ | *trnD-GUC*  *trnG-UCC*  *trnL-CAA^a^*  *trnP-UGG*  *trnS-GGA*  *trnV-UAC^b^* | *trnE-UUC*  *trnH-GUG*  *trnL-UAA^b^*  *trnQ-UUG*  *trnS-UGA*  *trnW-CCA* | *trnF-GAA*  *trnI-GAU^ab^*  *trnL-UAG*  *trnR-ACG^a^*  *trnT-GGU*  *trnY-GUA* |
|  | genes |  |  |  |  |  |
|  |  |  |  |  |  |  |
|  |  |  |  |  |  |  |
|  |  |  |  |  |  |  |
|  |  |  |  |  |  |  |
|  | Small Subunit | *rps2* | *rps3* | *rps4* | *rps7^a^* | *rps8* |
|  | of ribosome | *rps11* | *rps12^ac^* | *rps14* | *rps15* | *rps16^b^* |
|  |  | *rps18* | *rps19* |  |  |  |
|  | Large Subunit of | *rp12^ab^* | *rp114* | *rp116^b^* | *rp120* | *rp123^a^* |
|  | ribosome | *rp132* | *rp133* | *rp136* |  |  |
|  | DNA-dependent | *rpoA* | *rpoB* | *rpoC1^b^* | *rpoC2* |  |
|  | RNA polymerase |  |  |  |  |  |
| Genes for | Subunits of | *psaA* | *psaB* | *psaC* | *psaI* | *psaJ* |
| photosynthesis | photosystem I | *ycf3^c^* | *ycf4* |  |  |  |
|  | Subunits of | *psbA* | *psbB* | *psbC* | *psbD* | *psbE* |
|  | photosystem II | *psbF* | *psbH* | *psbI* | *psbJ* | *psbK* |
|  |  | *psbL* | *psbM* | *psbN* | *psbT* | *psbZ* |
|  | NADH | *ndhA^b^* | *ndhB^ab^* | *ndhC* | *ndhD* | *ndhE* |
|  | oxidoreductase | *ndhF* | *ndhG* | *ndhH* | *ndhI* | *ndhJ* |
|  |  | *ndhK* |  |  |  |  |
|  | Subunits of | *petA* | *petB^b^* | *petD^b^* | *petG* | *petL* |
|  | cytochrome | *petN* |  |  |  |  |
|  | Subunits of ATP | *atpA* | *atpB* | *atpE* | *atpF^b^* | *atpH* |
|  | synthase | *atpI* |  |  |  |  |
|  | Large subunit of | *rbcL* |  |  |  |  |
|  | Rubisco |  |  |  |  |  |
| Other genes | Maturase | *matk* |  |  |  |  |
|  | Envelope | *cemA* |  |  |  |  |
|  | membrane |  |  |  |  |  |
|  | protein |  |  |  |  |  |
|  | Subunit of | *accD* |  |  |  |  |
|  | acetyl-CoA |  |  |  |  |  |
|  | C-type | *ccsA* |  |  |  |  |
|  | cytochrome |  |  |  |  |  |
|  | synthesis gene |  |  |  |  |  |
|  | Protease | *clpP^c^* |  |  |  |  |
| Genes of unknown | Hypothetical | *ycf1^a^* | *ycf2 ^a^* | *ycf15 ^a^* |  |  |
| functions | chloroplast |  |  |  |  |  |
| Pseudogenes | Pseudogene | *ycf1^d^* | *rps19^d^* | *infA^d^* |  |  |

^a^ indicates the genes duplicated in the IR regions

^b^ indicates the genes containing a single intron.

^c.^indicates the genes containing two introns.

^d^ Pseudogene
