# Supplementary material for: Comparative Chloroplast Genomics of Gossypium Species: Insights Into Repeat Sequence Variations and Phylogeny
Source: Front Plant Sci. 2018 Mar 21;9:376. doi: 10.3389/fpls.2018.00376 (PMC5871733; doi:10.3389/fpls.2018.00376)
Supplement: TABLE S7 — Distribution of SSRs in cotton chloroplast genomes. [file Table_7.DOCX]

**Table S7** Distribution of SSRs in cotton chloroplast genomes.

| Species | Sub-groups | SSR Loci  (N) | P1 Loci  (N) | P2 Loci  (N) | P3 Loci  (N) | P4 Loci  (N) | P5 Loci  (N) | P6  Loci(N) | LSC | SSC | IRa | IRb |
| --- | --- | --- | --- | --- | --- | --- | --- | --- | --- | --- | --- | --- |
| *G. herbaceum subsp.africanum* | A1-a | 80 | 51 | 16 | 3 | 6 | 4 | / | 60 | 14 | 3 | 3 |
| *G. arboreum* | A2 | 77 | 48 | 16 | 3 | 6 | 4 | / | 58 | 13 | 3 | 3 |
| *G.hirsutum* race*latifolium* | AD1 | 70 | 44 | 15 | 2 | 6 | 2 | 1(CTTATT)3 | 54 | 12 | 2 | 2 |
| *G. tomentosum* | AD3 | 76 | 46 | 16 | 6 | 6 | 2 | / | 59 | 11 | 3 | 3 |
| *G. mustelinum* | AD4 | 73 | 43 | 16 | 4 | 6 | 4 | / | 56 | 11 | 3 | 3 |
| *G. darwinii* | AD5 | 71 | 43 | 16 | 4 | 6 | 2 | / | 55 | 12 | 2 | 2 |
| *G. hirsutum* | AD1 | 70 | 44 | 15 | 2 | 6 | 2 | 1(CTTATT)3 | 54 | 12 | 2 | 2 |
| *G. hirsutum*cultivar hainansijimia | AD1 | 72 | 44 | 16 | 3 | 6 | 2 | 1(CTTATT)3 | 56 | 12 | 2 | 2 |
| *G. barbadense* | AD2 | 74 | 48 | 15 | 3 | 6 | 2 | / | 59 | 11 | 2 | 2 |
| *G. barbadense* cultivar *Zhonghai7* | AD2 | 71 | 45 | 15 | 3 | 6 | 2 | / | 55 | 12 | 2 | 2 |
| *G. barbadense* cultivar *Yuanmou* | AD2 | 71 | 45 | 15 | 3 | 6 | 2 | / | 55 | 12 | 2 | 2 |
| *G. barbadense*cultivar*kaiyuan* | AD2 | 71 | 45 | 15 | 3 | 6 | 2 | / | 55 | 12 | 2 | 2 |
| *G. anomalum* | B1 | 60 | 39 | 14 | 2 | 5 | / | / | 45 | 11 | 2 | 2 |
| *G. capitis-viridis* | B3 | 61 | 40 | 14 | 2 | 5 | / | / | 47 | 12 | 1 | 1 |
| *G. sturtianum* | C1 | 57 | 35 | 10 | 3 | 6 | 1 | 2(TTAATA)4 | 42 | 13 | 1 | 1 |
| *G. robinsonii* | C2 | 67 | 44 | 11 | 5 | 6 | 1 | / | 52 | 11 | 2 | 2 |
| *G.nandewarense* | C1-n | 60 | 38 | 10 | 2 | 7 | 1 | 2(TTAATA)4 | 44 | 14 | 1 | 1 |
| *G. thurberi* | D1 | 81 | 54 | 14 | 2 | 7 | 2 | 2(ATAGAA)3 | 62 | 15 | 2 | 2 |
| *G. armourianum* | D2-1 | 78 | 59 | 11 | 2 | 5 | / | 1(ATAGAA)3 | 61 | 15 | 1 | 1 |
| *G. harknessii* | D2-2 | 76 | 54 | 11 | 2 | 6 | 2 | 1(ATAGAA)3 | 58 | 14 | 2 | 2 |
| *G. davidsonii* | D3-d | 75 | 51 | 11 | 2 | 7 | 4 | / | 53 | 16 | 3 | 3 |
| *G. klotzschianum* | D3-k | 74 | 48 | 11 | 3 | 8 | 4 | / | 53 | 15 | 3 | 3 |
| *G. aridum* | D4 | 77 | 55 | 10 | 2 | 8 | 2 | / | 60 | 15 | 1 | 1 |
| *G. raimondii* | D5 | 72 | 48 | 12 | 2 | 7 | 2 | 1(ATAGAA)3 | 54 | 14 | 2 | 2 |
| *G. gossypioides* | D6 | 74 | 51 | 12 | 2 | 6 | 2 | 1(ATAGAA)3 | 57 | 13 | 2 | 2 |
| *G.laxum* | D7 | 78 | 55 | 10 | 3 | 8 | 2 | / | 60 | 14 | 2 | 2 |
| *G.turneri* | D8 | 77 | 55 | 10 | 2 | 7 | 2 | 1(ATAGAA)3 | 58 | 15 | 2 | 2 |
| *G. harknessii* | D9 | 72 | 50 | 10 | 3 | 7 | 2 | / | 55 | 15 | 1 | 1 |
| *G. davidsonii* | D10 | 76 | 54 | 11 | 2 | 6 | 2 | 1(ATAGAA)3 | 58 | 14 | 2 | 2 |
| *G. schwendimanii* | D11 | 79 | 55 | 10 | 4 | 8 | 2 | / | 61 | 14 | 2 | 2 |
| *G. stocksii* | E1 | 66 | 46 | 11 | 1 | 8 | / | / | 52 | 12 | 1 | 1 |
| *G. somalense* | E2 | 76 | 55 | 10 | 4 | 7 | / | / | 56 | 16 | 2 | 2 |
| *G. areysianum* | E3 | 77 | 56 | 10 | 4 | 7 | / | / | 57 | 16 | 2 | 2 |
| *G. incanum* | E4 | 67 | 47 | 11 | 1 | 8 | / | / | 51 | 12 | 2 | 2 |
| *G. longicalyx* | F1 | 87 | 54 | 14 | 5 | 7 | 4 | 3(TTAAAT)6 | 63 | 16 | 4 | 4 |
| *G. bickii* | G1 | 58 | 38 | 10 | 2 | 7 | 1 | / | 44 | 12 | 1 | 1 |
| *G. australe* | G2 | 78 | 58 | 10 | 3 | 7 | / | / | 64 | 12 | 1 | 1 |
| *G. populifolium* | K2 | 72 | 51 | 11 | 2 | 7 | 1 | / | 58 | 12 | 1 | 1 |
| **Total** |  | 2751 | 1836 | 475 | 106 | 249 | 67 | 18 | 2101 | 502 | 74 | 74 |
