# Supplementary material for: Comparative Chloroplast Genomics of Gossypium Species: Insights Into Repeat Sequence Variations and Phylogeny
Source: Front Plant Sci. 2018 Mar 21;9:376. doi: 10.3389/fpls.2018.00376 (PMC5871733; doi:10.3389/fpls.2018.00376)
Supplement: TABLE S11 — Likelihood ratio test (LRT) of the variable ω ratio under different models. [file Table_11.DOCX]

| gene | comparisons | 2Δ*L* | *df* | *P* |
| --- | --- | --- | --- | --- |
| *atpB* | M0 vs M3 | 108.444588 | 4 | 1.34E-21 |
|  | M1 vs M2 | 65.598394 | 2 | 6.05E-15 |
|  | M7 vs M8 | 66.488924 | 2 | 3.22E-15 |
| *atpE* | M0 vs M3 | 66.258896 | 4 | 1.44E-10 |
|  | M1 vs M2 | 30.493304 | 2 | 2.29E-05 |
|  | M7 vs M8 | 30.573052 | 2 | 2.19E-05 |
| *petB* | M0 vs M3 | 136.799504 | 4 | 4.72E-30 |
|  | M1 vs M2 | 94.965894 | 2 | 1.41E-24 |
|  | M7 vs M8 | 94.039994 | 2 | 2.95E-24 |
| *petD* | M0 vs M3 | 258.238812 | 4 | 2.90E-72 |
|  | M1 vs M2 | 237.411206 | 2 | 6.37E-82 |
|  | M7 vs M8 | 253.445084 | 2 | 4.20E-89 |
| *ccsA* | M0 vs M3 | 31.24154 | 4 | 1.45E-03 |
|  | M1 vs M2 | 19.6172 | 2 | 4.32E-03 |
|  | M7 vs M8 | 31.01833 | 2 | 1.73E-05 |
| *cemA* | M0 vs M3 | 338.16632 | 4 | 1.06E-103 |
|  | M1 vs M2 | 293.840564 | 2 | 1.15E-107 |
|  | M7 vs M8 | 294.7275 | 2 | 4.44E-108 |
| *rbcL* | M0 vs M3 | 55.49093 | 4 | 3.77E-08 |
|  | M1 vs M2 | 29.301738 | 2 | 4.27E-05 |
|  | M7 vs M8 | 37.82115 | 2 | 3.97E-07 |
| *rps2* | M0 vs M3 | 25.522372 | 4 | 1.03E-03 |
|  | M1 vs M2 | 18.834218 | 2 | 6.03E-03 |
|  | M7 vs M8 | 14.301754 | 2 | 3.60E-03 |
| *rps3* | M0 vs M3 | 337.276822 | 4 | 2.45E-103 |
|  | M1 vs M2 | 301.085182 | 2 | 4.65E-111 |
|  | M7 vs M8 | 328.8237 | 2 | 3.16E-124 |
| *ycf1* | M0 vs M3 | 142.552376 | 4 | 7.55E-32 |
|  | M1 vs M2 | 125.559598 | 2 | 1.20E-35 |
|  | M7 vs M8 | 104.326948 | 2 | 7.24E-28 |

**Table S11.** Likelihood ratio test (LRT) of the variable ω ratio under different models.
